# Editorial: 3D-printed biomaterials in osteochondral repair

**DOI:** 10.3389/fbioe.2022.983945

**Published:** 2022-08-16

**Authors:** Changchun Zhou, Lan Li, Xiaofeng Jia, Lei Zhang

**Affiliations:** ^1^ National Engineering Research Center for Biomaterials, College of Biomedical Engineering, Sichuan University, Chengdu, China; ^2^ Nanjing Drum Tower Hospital, Nanjing, China; ^3^ Department of Neurosurgery, Orthopaedics, and Anatomy & Neurobiology, School of Medicine, University of Maryland, Baltimore, MD, United States; ^4^ Department of Orthopaedics, The First Affiliated Hospital of Wenzhou Medical University, Wenzhou, China

**Keywords:** 3D printing, bone repair, chondral regeneration, tissue engineering, regenerative medicine

Osteochondral lesion appears a significant challenge for orthopaedic surgeons worldwide, and is often associated with multiple factors such as acute trauma or degenerative processes. To overcome major challenges facing massive bone or cartilage defects, three-dimensional (3D) printing technology has been developed rapidly in recent years, and shows broad prospects in osteochondral repair, leading to favorable clinical outcomes. Currently, an increasing number of novel nanocomposite biomimetic biomaterials, based on 3D printing technology, have shown promise for bone and cartilage engineering. Therefore, to highlight the latest progress, this Research Topic aims to provide an update on the novel 3D printing technologies and biomaterials.

Here, the Research Topic collected a total of 14 papers, including 13 original articles and one review, which cover a wide range of the novel 3D-printed biomaterials, from the design, preparation, evaluation to preclinical application, and summarize the significant progress and applications of 3D printing technology for bone-cartilage interface regeneration. The papers in this topic are briefly shared below.

To overcome the limitations of mismatched mechanical and unsatisfactory biological properties of PLGA in bone tissue engineering, Liu et al. added calcium sulfate and prepared a new customized-designed 3D porous PLGA/CaSO_4_ scaffold. These 3D-printed scaffolds exhibited not only satisfactory biocompatibility, but enhanced mechanical and biological properties as well. These customized fabricated PLGA/CaSO_4_ scaffolds show great potential for precisely repairing irregular load-bearing bone defects.

Recommendations for surgical fixation of tibiofibular syndesmosis injuries are challenging task for many clinical orthopedists due to international consensus has not been published for the optimal treatment of the injury. Zhang et al. created a 3D printed navigation template for a precise bone tunnel and a novel adjustable EndoButton fixation (NAE) for the ideal treatment. The accuracy of the 3D-printed navigation template and the biomechanical performance of the NAE technique have been well evaluated and explored. The 3D printing technology application may become beneficial and favorable for locating and preparing the bone tunnel.

The rational structural design of the trabecular porous scaffold is a key point for the satisfactory efficacy of the implant. Chao et al. prepared porous scaffolds with biomimetic potential based probability balls and the Voronoi–Tessellation approach. These 3D-printed biomaterials appeared trabecular-like porous microstructure and tissue morphology, which can address stress shielding and bone ingrowth in existing biomimetic bone structures. In addition, the scaffolds could also promote cell adhesion, migration, and eventual new bone attachment. Based on the existing research, animal implantation experiments should be carried out to verify the biological properties of porous micro-structures.

Chai et al. created a photo-crosslinked composite bioactive scaffold based on GelMA-BMSCs-BMP2. The composite bioactive scaffolds effectively promoted the osteogenic differentiation and bone tissue regeneration of BMSCs, and the biosafety of the composite scaffold was verified *in vivo* and *in vitro* experiments. Therefore, BMP2 and BMSCs in GelMA hydrogel scaffolds showed good synergistic effects on encouraging bone defect repair, and thus offered a new option for the treatment of irregular bone deformities.

Precartilaginous stem cells (PCSCs) can be used as seed cells and incorporated with bioactive scaffolds for reconstructive tissue therapy of bone defects, and iron oxide nanoparticles (IONPs) can enhance osteogenic differentiation potential by modulating the fate of PCSCs. Liao et al. revealed osteogenesis of PCSCs induced by IONPs, as evidenced by enhanced ALP activity levels, mineralized matrix nodules, and osteogenesis-related gene expression. In addition, the IONPs-labeled PCSCs-incorporated polymeric printable network (IPN) hydrogel was of great printability, biosafety, and improvement of cell spreading and proliferation. Therefore, PCSCs-based scaffolds could enrich the stem cell-based therapeutic strategies for bone tissue regeneration.

For osteochondral damage, the pH value change of the damaged site will influence the repair efficacy of the patient. Long et al. constructed a vitro model which provided a simple and practical platform to evaluate the influence of the acid-base effect. The study prepared a porous silica-based solid-acid catalyst material by additive manufacturing technology and the results showed excellent catalytic performance. The catalytic strategy by constructing a *vitro* model supplies an alternative way in environment evaluation of osteochondral repair and it also shows potential for the enzymatic catalytic research during the body’s metabolism process in the future studies.

Besides cartilage itself, the research of cartilage regeneration should additionally consider the subchondral bone, which also plays a critical role in its process. Therefore, the integrated repair of bone-cartilage may be the future direction of tissue engineering scaffolds. Xu et al. summarized the latest and significant developments in bone-cartilage interface regeneration using 3D printing techniques. The recent progress of bioinks, scaffold structures, bioactive factors, and bioreactors were detailed descripted in this review. In addition, the potential prospects and challenges of 3D printing techniques in bone-cartilage tissue engineering were also particularly exhibited.

Based on the concept of integrated repair of bone-cartilage, Yuan et al. designed a novel polyetheretherketone (PEEK) scaffold. This porous non-degradable scaffold was modified using the chitosan, mesoporous silica nanoparticles, platelet-derived growth factor BB, kartogenin, and polydopamine. The composite scaffold gained both osteogenic and chondrogenic capacity. It could promote cell migration and enhance chondrogenic differentiation of BMSCs *in vitro*, and facilitate cartilage regeneration *in vivo*. This study reveals a new therapeutic concept of porous bioactive prosthesis for osteoarticular lesions.

Polycaprolactone (PCL) is another generally used biomaterial for fabricating rigid scaffold framework. Wu et al. explored the feasibility of replacing the decalcified bone matrix (DBM) framework with a PCL framework. They found that the inflammatory response was reduced and the mature cartilage can be constructed by this scaffold. The components of the regenerated cartilage were closer to the natural cartilage. The 3D-printed PCL framework method shows the unique advantage in clinical cartilage regeneration due to the features of customizable shape design, mechanical strength control, and standardized production.

For the purpose of mimicking the natural bone components and promoting bone regeneration using a cell-free scaffold, Tang et al. developed a photocrosslinked interpenetrating polymeric network (IPN) bioink. Through the introducing of platelet-rich plasma and biological mineralization, the osteogenic bioactivities can be effectively enhanced. The *in vivo* study has proved that this IPN hydrogel can significantly accelerate bone regeneration and can be regarded as a promising strategy in the fabrication of 3D-printed bone scaffolds.

The metal-based 3D printed scaffolds are one of the options for bone regeneration. Yang et al. employed laser additive manufacturing technique to fabricate a Fe/calcium chloride (CaCl_2_) based composite scaffold. Compared to traditional Fe-based scaffold, the addition of CaCl_2_ could strongly improve the stability and corrosion resistance. The surface topography was also affected by the release of chloride ion during the manufacture and corrosion process. The osteogenic ability was enhanced due to the existence of Ca ion. This composite iron combination demonstrated admirable prospects in bone scaffold fabrication.

The organic combination of biomimetic materials and stem cells offers new strategies for bone tissue engineering, and the fate of stem cells is closely related to their extracellular matrix (ECM) properties. Li et al. prepared a photocrosslinked biomimetic methacrylated gelatin (Bio-GelMA) hydrogel scaffold loaded with BMSCs to examine the therapeutic effects of ECM-loaded cells in a 3D environment simulated for segmental bone defects. The results showed that the BMSC-carrying GelMA hydrogel scaffold has good mechanical properties and biological compatibility. In addition, the bio-GelMA scaffold can be used as a cell carrier of BMSC to promote the regeneration of bone and blood vessels, to improve the mechanical strength of bone defects, and to effectively promote the repair of bone defects. Therefore, the BMSC-carrying GelMA hydrogel scaffold offered a new option for the treatment of segment bone defects.

The micro geometry and surface roughness of PCL scaffolds may change the biocompatibility and bioactivity of the scaffolds during the SLS process. However, it is still unknown how SLS process parameters affect the surface roughness of PCL scaffolds and the relationship between roughness and biocompatibility of scaffolds. Han et al. prepared five PCL scaffolds with different laser power and scanning speed. Furthermore, the study calculated the energy density range (Ed1-Ed3) suitable for PCL sintering by using the energy density model (EDM) combined with the thermodynamic properties of PCL powder. As the result, the dense and smooth surface of scaffolds had poor cytocompatibility, while the low energy density (Ed1) resulted in weak mechanical properties, but the rough surface caused by incomplete sintered PCL particles facilitated the cells adhesion and proliferation. Therefore, the surface roughness and related biocompatibility of PCL bone scaffolds should be considered in energy-density-guided SLS parameters optimization.

Yang et al. fabricated porous DLP-printed BCP bioceramic scaffolds coated with polydopamine/BMP-2. The scaffolds with superhydrophilicity and BMP-2 delivery and sustained-release abilities exhibited an interconnected porous structure and acceptable compressive strength matched with load-free cancellous bone. In addition, the BMP-2/PDA-BCP scaffold presented favorable effects on the adhesion, proliferation, osteogenic differentiation, and mineralization of BMSCs. *In vivo* results of SD rats demonstrated the PDA coating induced cell aggregation nearby the coating and continuous lamellar new bone formation within the scaffold. This study provided a promising strategy to fabricate bone substitute scaffolds for enhanced bone regeneration for bone defects in demand for high precision and small size.

